# Temperature-Driven Morphological and Microstructural Changes of Gold Nanoparticles Prepared by Aggregation from the Gas Phase

**DOI:** 10.1021/acsomega.5c02149

**Published:** 2025-05-20

**Authors:** Tereza Košutová, Zdeněk Krtouš, Jaroslav Kousal, Ondřej Kylián, Jan Hanuš, Lidia Martínez, Yves Huttel, Daniil Nikitin, Pavel Pleskunov, Hynek Biederman, Lukáš Horák, Milan Dopita

**Affiliations:** † Department of Condensed Matter Physics, 37740 Faculty of Mathematics and Physics, Charles University, Ke Karlovu 5, Prague, 121 16, Czech Republic; ‡ Department of Macromolecular Physics, Faculty of Mathematics and Physics Charles University, V Holešovičkách 2, Prague 180 00, Czech Republic; § Instituto de Ciencia de Materiales de Madrid, Consejo Superior de Investigaciones Científicas (CSIC), C/Sor Juana Inés de la Cruz, 3, Madrid 28049, Spain

## Abstract

The effect of annealing on the thin layers of gold and gold nanoparticles in air was studied by statistically relevant X-ray scattering methods. The nanoparticle behavior is found to depend on the substrate coverage and annealing temperature. During annealing up to 450 °C, the size of single-crystalline nanoparticles gradually increases through the process of Ostwald ripening, while the density of crystallographic defects decreases slightly. An abrupt change occurs above 450 °C, whereas no significant evolution is observed for the less covered sample; at the sample with more material, the nanoparticles coalesce, and their shape becomes more rounded by further annealing. Only after the spheroidization is completed do the sizes of crystallites follow the nanoparticle size growth. Comparison with the thin continuous gold layer shows that the healing of the crystallographic defects, i.e., microstrain and stacking faults, takes place at significantly lower temperatures if the material is evenly distributed on the silicon substrate surface. However, annealed nanoparticle layers provide a much narrower particle size distribution when compared to a dewetted gold thin layer. At around 800 °C, the alignment of the gold crystal structure toward the substrate is detected, and it changes from the random distribution of the atomic planes given by the random initial orientation of deposited nanoparticles. Another interesting phenomenon occurs for annealing above 1000 °C; for the nanoparticle layers, the smallest nanoparticles evaporate, leaving holes in the SiO_2_ surface layer.

## Graphical abstract

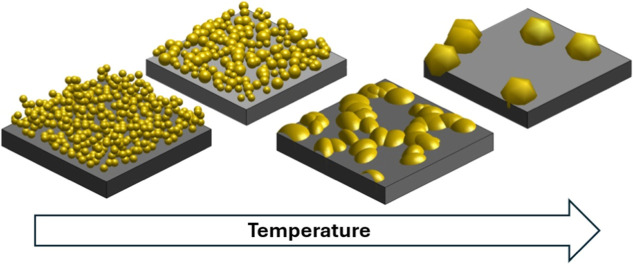

## 1 Introduction

Despite its high price, gold remains one of the most demanded materials. The use of Au was traditionally connected to its outstanding properties such as its inertness, biocompatibility, aesthetics, and corrosion resistance. However, with the rapid development in nanotechnology and the exploration of unique photonic, electronic, catalytic, magnetic, and therapeutic characteristics of nanosized gold (e.g., nanorods, nanoshells, nanocages, or nanoparticles), gold has become an almost irreplaceable material in a wide range of modern applications, such as nanomedicine,^1^ drug-delivery,^2^ imaging,^3^ photothermal therapy,^4^ sensing,^5^ and catalysis,^6^ or as active component in light absorbers^7^ or light-to-heat convertors.^8^ In most of the aforementioned examples, the performance of Au nanomaterials is strongly influenced not only by their shape and size but also by the number of crystal defects^9^ or the amount of lattice strain.^10^ In addition, in some of the applications, the Au nanomaterials are exposed to elevated temperatures (e.g., catalysis,^11^ laser desorption-ionization mass spectrometry,^12^ or photothermal therapy^13^). However, higher temperatures may lead to the sintering or restructuring of Au nanomaterials, and thus their functional properties may be compromised or altered after the heating. Because of this, detailed knowledge of the heat-induced changes in Au nanomaterials is of paramount importance and has been the subject of numerous investigations. Nevertheless, the performed theoretical and experimental studies are often limited in different aspects. Concerning the theoretical models, although they allow for predicting the changes in the crystalline structure of nanoparticles (NPs),^14,15^ the mechanism of sintering,^16^ or growth and shape changes of Au NPs, the computing time restricts them to study systems that involve a small number (usually only two) of tiny (typically around few nm) NPs. In addition, the studied NPs are often modeled as self-standing, i.e., without interactions with the substrate, which does not reflect the real situation. Experimentally, mainly by the in situ characterization of Au NPs upon their heating by transmission electron microscopy, different stages during the heating were directly observed—neck formation between individual nanoparticles,^17^ structure alignment,^18^ spheroidization of nanoparticles, and the appearance of facets.^19^ However, the measurements were performed under high-vacuum conditions and provided precise but highly localized pictures for a low number of nanoparticles, which is not sufficient for the understanding of the behavior of systems composed of a larger number of Au NPs.

The main goal of our study is to analyze the real and complex system of Au nanoparticle films under mid- and high-temperature conditions in a statistical manner using morphological and crystallographic characterization methods (scanning electron microscopy and X-ray scattering methods), allowing the precise determination of the heat-induced genesis of the morphology and microstructure of gold NPs. In our study, we investigated samples with two different coverages of nanoparticles on the substrates prepared employing a magnetron-based gas aggregation source (Haberland’s type^20^). It leads not just to the smaller/larger volume of material but also to the additional number of interactions between nanoparticles on a more densely covered substrate that, as will be shown, influences the properties of heated Au NPs. For the comparison of the obtained microstructural parameters, the thin gold layer was analyzed in the same manner.

## 2 Experimental Methods

### 2.1 Samples Preparation

The Au nanoparticle films deposition was carried out using a magnetron-based gas aggregation source NC200U–B from Oxford Applied Research Ltd.^21^ loaded with an Au target (99.95% purity) and operated at 60 W with a flux of argon in the aggregation zone of 60 standard cubic centimeters per minute. The cluster source was connected to a deposition chamber provided with a fast entry load lock. Both the cluster source and deposition chamber had a base pressure in the low 10^–10^ mbar range. All deposits were performed on silicon wafers with their native oxide layer. The samples with two different surface coverages were prepared by adjusting the deposition time. The deposition time for the higher coverage was 6 times longer and so was the number of NPs. These coverages will be for simplicity denoted as low- and high-coverage, respectively, through the subsequent text.

The Au thin film was prepared by DC magnetron sputtering in an argon atmosphere using a 2 in., water-cooled planar magnetron equipped with an Au target. The deposition conditions (magnetron current 100 mA, pressure 3 Pa, deposition time 180 s) were adjusted in a way to produce 15 nm thick Au films. The resulting 15 nm thick Au layer has an equivalent amount of material as the high-coverage nanoparticle sample.

In order to investigate the temperature-induced changes in Au NPs, the samples were annealed in the heating chamber at atmospheric pressure with a heating rate of approximately 15 °C per minute. After reaching the targeted temperature, samples were held for 10 min at the temperature followed by a slow cooldown to room temperature.

### 2.2 Samples Characterization

Our goal, i.e., the description of the temperature-induced morphological and microstructural changes, is achieved by combining multiple characterization techniques.

The morphology of nanoparticles and Au thin film was evaluated by using a JSM-7200F (JEOL) scanning electron microscope operated in the secondary-electron mode.

Small-angle X-ray scattering (SAXS) measurements were performed in transmission geometry for the samples of nanoparticles using a Xenocs Xeuss 2.0 SAXS instrument equipped with a Mo X-ray source (Mo Kα radiation, λ = 0.07107 nm), parallel beam collimating single reflection multilayer mirror, two sets of scatterless slits in the primary beam, and Dectris Pilatus 200k detector. The sample-to-detector distance was 2500 mm, allowing the accessible q-range of 0.01–0.3 Å^–1^. Measured images were azimuthally integrated, corrected for the background, and normalized. Resulting 1D SAXS curves were fitted to obtain the distribution of nanoparticle sizes. The form factor for spherical particles (Spheroid) was used for the fitting by the Irena package.^22^ SAXS data were fitted by the model of the unimodal size distribution of the diluted spheres.

The ex situ XRD characterization of the samples was done on a Rigaku SmartLab diffractometer equipped with a 9 kW copper rotating anode X-ray source (CuKα radiation, λ = 0.15418 nm), a parabolic multilayer mirror in the primary beam, a set of axial divergence eliminating Soller slits in both incident and diffracted beam (acceptance 5°), a parallel beam Soller slit collimator (acceptance 0.5°), and a HyPix-3000 2D hybrid pixel single photon counting detector in the diffracted beam. The constant incidence angle of the primary beam ω = 0.6° was used for the measurements. Measured diffraction patterns were fitted using the whole powder pattern refinement method (Rietveld method). The computer program MStruct^23^ was used for the fitting and analysis of the crystalline phases present in the investigated samples. Using MStruct software, the phase composition, lattice parameters, sizes of coherently diffracting domains (crystallite sizes), microstrain, and stacking faults present in the crystalline phases were determined. In the case of the density of stacking faults, these were used as fitting parameters in the program MStruct, into which the theory of the influence of stacking faults on XRD patterns is incorporated according to ref 24. The type of deformation stacking faults was assigned and checked by a procedure described in ref 25, based on the shifts of the individual peaks from the diffraction pattern. Also, incorporating the microstructural defect parameters into the model significantly improves the fit; see goodness-of-fit values in Table S1.

The in situ XRD measurement of Au nanoparticle films during their heat treatment was done on a PANalytical X’Pert MRD diffractometer equipped with a copper line focused X-ray source (Cu Kα radiation, λ = 0.15418 nm), a parabolic X-ray mirror producing incident parallel beam, a set of axial divergence eliminating Soller slits in both incident and diffracted beam (acceptance 2.3°), a parallel plate collimator (acceptance 0.27°), and a proportional point detector. The constant incidence angle of the primary beam ω = 1.25° was used for the measurements. During the heating experiment, the sample was placed in the heating stage DHS 1100 (Anton Paar) with a graphite dome. The heating procedure consists of an increase of temperature by steps 50 °C from room temperature to 950 °C. Above 850 °C, the sample was cooled down to examine changes in the volume of the crystalline phase. After the set temperature was reached, the fast measurement (11.5 min) started and was repeated 10 times for each temperature, which was kept constant during the measurements.

The measurement of the X-ray reflectivity was used to determine the thickness of the gold thin layer. The preferred orientation of crystallites on the substrate was investigated by pole figure measurements and in situ by χ scans for the high-coverage sample. All in situ annealing experiments were performed in an air atmosphere.

## 3 Results and Discussion

### 3.1 As-Deposited Au Samples

The top-view scanning electron microscopy (SEM) images of the as-prepared low- (6 times less material) and high-coverage (equivalent amount of material as for the 15 nm thick Au layer) samples and the gold thin layer are displayed in Figure 1a. The samples of two different coverages contain nanoparticles differing only in their amount, while their sizes are independent of the surface coverage. This is confirmed by SAXS (see Figure 1b), which revealed that Au NPs have a mean of the volume-weighted size distribution of 32 ± 2 and 29 ± 2 nm for high and low coverage, respectively. The size distribution of nanoparticles was found to be log-normal, as is expected for nanoparticles prepared by aggregation from the gas phase.^26^ A similar distribution of nanoparticle sizes was observed for the low-coverage sample also by SEM (comparison shown in Figure 1c). For this analysis, individual nanoparticles were identified and measured in SEM images, so it could only be performed with good confidence on the low-coverage sample. The mean size extracted from the obtained size distribution using log-normal fitting is 22 ± 4 nm. To compare this value to the results from SAXS, the size distribution was converted into log-normal volume-weighted and that possesses the mean value of 26 ± 5 nm.

**Figure 1:**
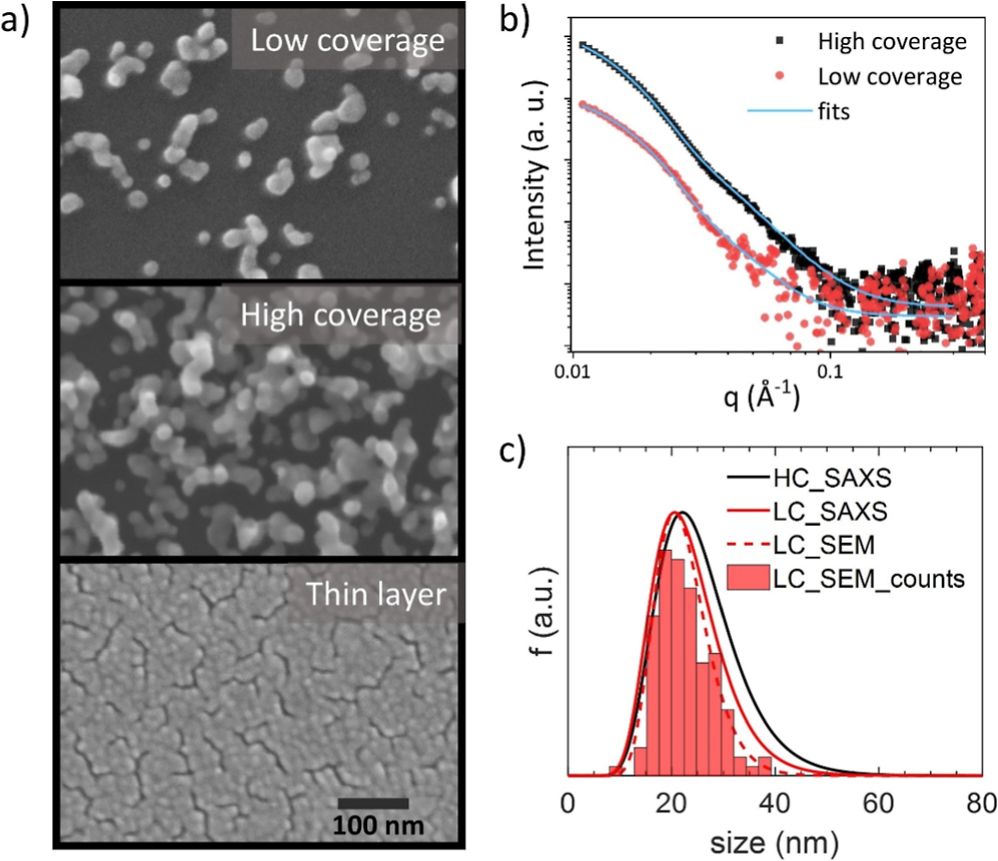
(a) SEM images of Au nanoparticles and thin film layers. (b) Measured 1D SAXS curves of Au nanoparticles films with low and high coverages including the fit using a model of unimodal size distribution of diluted spheres. (c) Number-weighted size distributions of nanoparticles obtained from SAXS and SEM measurements for high-coverage (HC) and low-coverage (LC).

In addition, a 15 nm thick gold layer sample, which was prepared to compare the microstructural changes during annealing, was found to contain cracks on the surface originating from the later stage of Au island growth on the upper native SiO_2_ layer. Still, the overall roughness determined by an X-ray reflectivity method together with the thickness of the layer (see the fitted data in Figure S1 in the Supporting Information) is relatively low, around 1.3 nm.

The deposited gold in different forms has the same face-centered cubic (fcc) crystal structure (see diffraction patterns in Figure 2a), i.e., the most common structure for gold.^27^ The lattice parameters obtained for Au NPs, i.e., 4.080(2) Å, are within the standard deviation, in agreement with the lattice parameter of bulk gold 4.07858(2) Å.^28^ This agreement points out that the sizes of nanoparticles are already large enough to minimize the effect of the surface stress leading to commonly observed lattice contraction for smaller metallic nanoparticles.^29^

**Figure 2:**
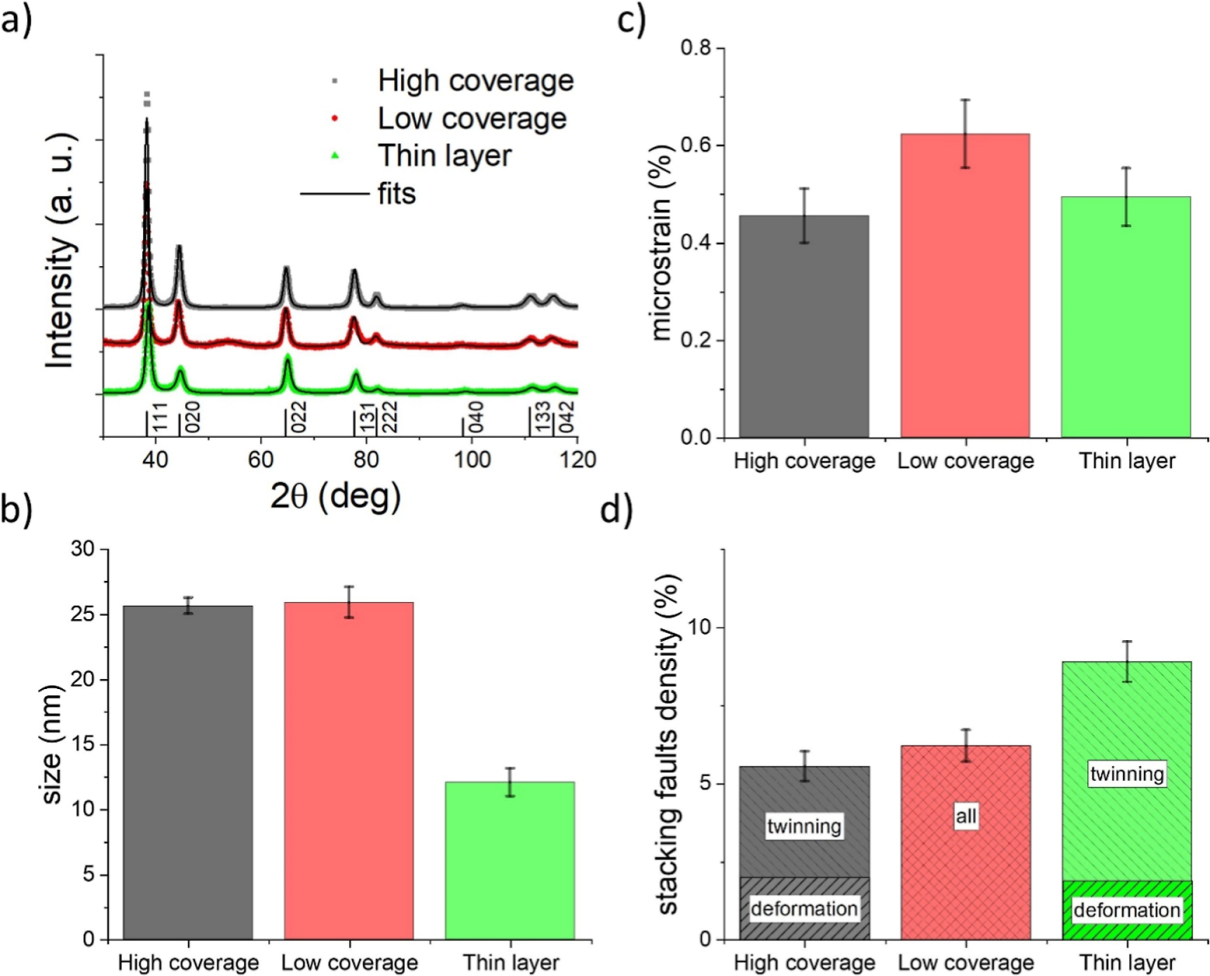
(a) XRD patterns, (b) size of crystallites, (c) microstrain, and (d) density of stacking faults measured on deposited gold in different forms extracted from XRD analysis.

According to XRD measurements (Figure 2b), the mean size of crystallites, i.e., the coherently diffracting domains, of as-deposited Au NPs is around 26 nm, i.e., approximately 4 nm smaller than the mean size of NPs detected by SAXS. This apparent discrepancy relates to the different working principles of SAXS and XRD; while SAXS is sensitive to the contrast of electron density, i.e., the electron density difference between the vacuum and gold NPs in our case, and thus sees the whole nanoparticle size, XRD gives information about the crystalline parts of NPs. Described observation, together with the absence of visible facets for as-deposited nanoparticles, suggests that nanoparticles consist of a crystallographically less ordered surface.

Unlike the cases of Au nanoparticle films, the lattice contraction effect is observed for the thin Au layer, where the lattice parameter, 4.0744(8) Å, is contracted for around 0.1% as compared to bulk gold. This effect can be ascribed to much smaller crystallites in the case of sputtered Au film as compared to Au NPs, which were found to be approximately 12 nm, i.e., comparable to the overall thickness of the Au film.

In addition to the information concerning the size of crystallites, the analysis of the diffraction patterns can also reveal the deviations from the perfect structure such as by microstrain or defects in the sequence of the atomic layers, stacking faults. Microstrain in the nanoparticles, defined as a relative deviation in interplanar spacing d concerning the relaxed one— $\frac{\Delta d}{d}$ , originates in structural disorder, i.e., vacancies, interplanar distance changes due to surface strain, dislocations, stacking faults, etc. The dominance of the type of structural defects depends on the crystallographic space group, size of nanoparticles, and preparation process. For nanoparticles synthesized using a gas aggregation source, the microstrain is probably mainly introduced to the nanoparticle structure during the formation process in the aggregation chamber, and the amount of microstrain is significant. Some microstrain may be partially released when nanoparticles are in contact. Because of this, we observe slightly higher microstrain in the less covered sample (0.63%) as compared to the sample with higher surface coverage of Au NPs (0.45%), as depicted in Figure 2c. In the case of the sputtered gold layer, the microstrain in the crystal structure rises during the deposition process,^30^ and the amount (0.49%) is comparable to the microstrain in the Au nanoparticle sample of high surface coverage.

The peak broadening in the diffraction patterns is caused by both crystallite sizes and the presence of microstrain. To distinguish between these two origins of peak broadenings, the size of crystallites and microstrain, the MStruct program was used. The values obtained from fitting were also checked by the standard Williamson–Hall plot method. For this method, discrepancies were observed, which indicate the presence of another type of disorder in the crystal lattice: the stacking faults, i.e., defects in a sequence of the atomic layers. These are incorporated as another refined microstructural parameter in the MStruct program.

Two types of stacking faults often occur in NPs consisting of fcc metals. Let us consider the default sequence of the fcc structure to be ABCABCABC, where A, B, and C represent the {111} planes. One faulty positioned (or missing) layer beyond which the sequence continues in the same order causes deformation stacking fault: the order is then ABCABABC (here, one layer is missing). The second type of stacking fault is called the twinning, beyond the missing plane, the order of the sequence is reversed, ABCABACBA. In our samples, both deformation and twinning stacking faults were detected. The stacking faults are formed during the preparation process, which is far from thermodynamic equilibrium. The atoms and smaller clusters aggregate and grow by collisions inside the aggregation chamber. In the theory proposed for the case of nanowires, it was shown that the twinning leads to the reduction of the total surface energy.^31^ Furthermore, the computation of thermodynamical models for nanocrystals has shown that the shape of a decahedron containing five twin planes in a cyclic configuration has the lowest free energy in the case of small particles (<10 nm) and the truncated octahedral structure has the lowest energy for the larger ones.^32,33^ So even the theoretical calculations of free-standing NPs predict the presence of planar defects within NPs. This is because of the extremely low stacking faults energy for gold.^34^ The refined stacking fault density is presented in Figure 2d. It can be seen that for samples with NPs, the stacking fault density is around 6%, while for the gold layer, the faults density is even higher, reaching almost 9%.

In the cases of higher coverage samples and Au thin films, twinning and deformation faults can also be distinguished from the recorded XRD patterns. For the nanoparticle film, the twinning faults density is around 3.5% while the deformation faults density is considerably lower and reaches the value of 2% (Figure 2d). The lower density of deformation stacking faults in the Au NPs as compared to twinning faults is caused by the approximately 50% lower energy of twinning stacking faults than the energy of the deformation faults when the lattice relaxation effect is neglected.^35^ This effect, i.e., the higher abundance of twinning faults, is even enhanced in the case of the Au thin film (Figure 2d).

Finally, the as-prepared Au NPs deposited in a soft-landing regime do not undergo structural rearrangement upon impact due to their relatively large size^36,37^ and are crystallographically randomly oriented on the substrates. Contrary to that, the magnetron-sputtered gold layer is crystallographically textured—the grains having the {111} plane parallel to the surface are more frequent in the layer, as already observed.^30^ This is caused by the low energy of the {111} surface and the successive formation of the gold layer already on the substrate.

### 3.2 Morphogenesis and Microstructure of Heat-Treated Samples

The morphology of Au nanoparticles was directly observed by SEM after the samples were annealed in air. Figure 3 presents the SEM top-view micrographs of as-deposited and annealed samples and corresponding mean sizes of Au NPs determined by SAXS. Two or three different temperature regions may be distinguished, depending on the surface coverage with Au NPs or a magnetron sputtered layer.

**Figure 3:**
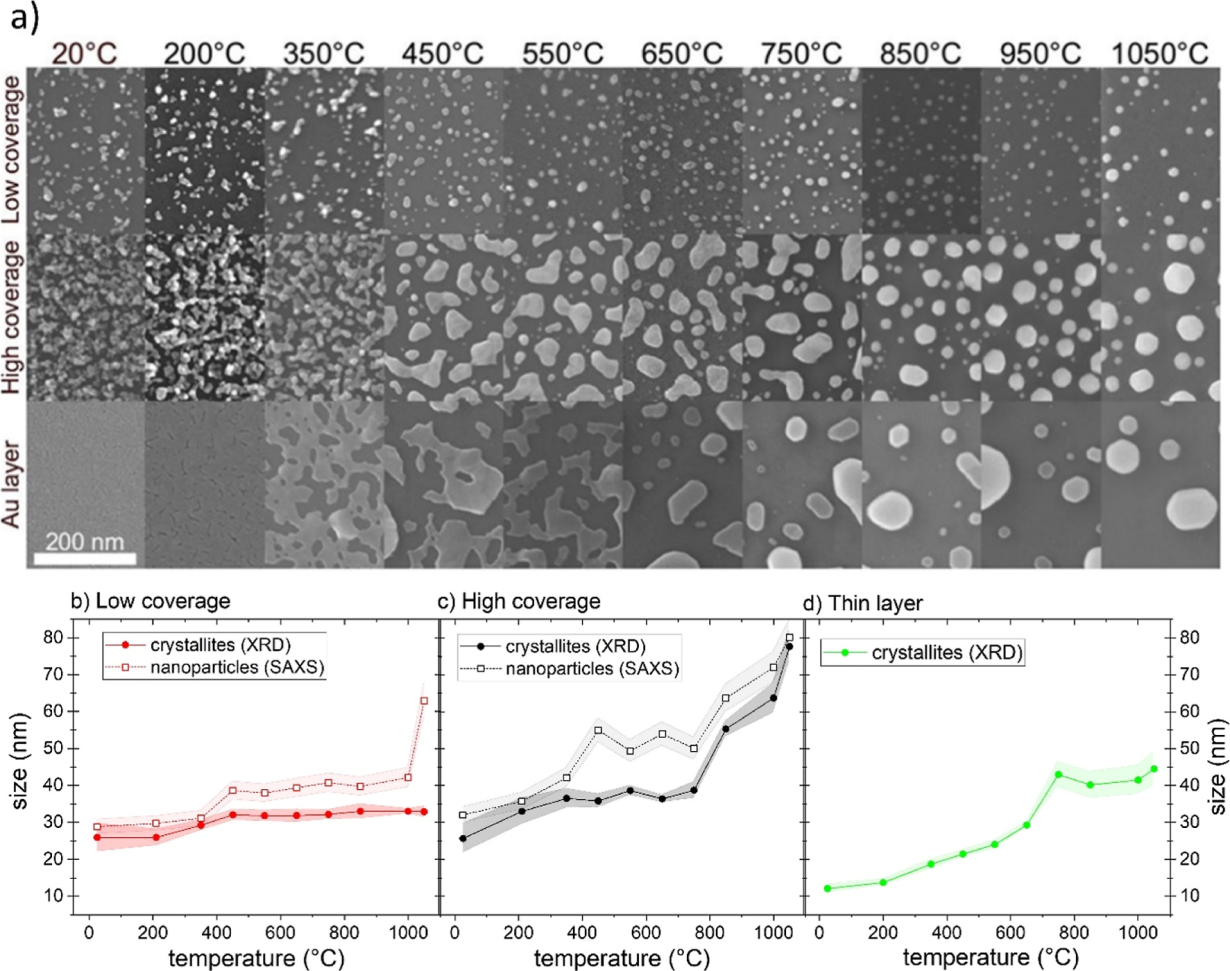
(a) Top-view SEM micrographs measured ex situ after each annealing temperature for both coverages and a thin gold layer. Sizes of nanoparticles and crystallites as a function of temperature for (b) low-coverage and (c) high-coverage samples. (d) Evolution of sizes of crystallites with temperature as measured by XRD for the Au thin film. The standard deviations are depicted as shaded areas.

In the first temperature region, which is common for both Au NPs samples and goes approximately up to 450 °C, an increase in the mean size of Au NPs was observed by means of SAXS (Figure 3b and c). Furthermore, this increase in the size of NPs was found to go hand in hand with the gradual increase in the size of crystallite sizes measured by XRD (Figure 3b and c). The growth of NPs in this temperature region arises from the Ostwald ripening process—particles increase in size through the transfer of atoms/molecules from noncrystalline parts/smaller particles to larger ones. Therefore, adatoms are incorporated into the crystal structure of the NPs to which they are attached. For high coverage, the crystallites are enlarged by this process to 36 nm. Meanwhile, for the low-coverage samples, the size of crystallites after heating to 450 °C is slightly smaller, approximately 32 nm. This difference is caused by the lower amount of material on the substrate for low-coverage samples.

From 450 °C on, the second temperature region starts and differs for low- and high-coverage samples. While in the case of low-coverage samples, no significant changes are observed, apart from the sintering of closely spaced particles, substantial changes in the Au morphology can be observed for high-coverage samples (Figure 3a). The growth mechanism in this midtemperature region changes from Ostwald ripening to coalescence of neighboring particles: the delivered energy in this temperature zone is high enough for the formation of nonspherical assemblies. Particles forming these assemblies are connected through the necks (see top-view SEM images presented in Figure 3a), which are well described in the literature for early stages of nanoparticle coalescence.^38^ At this moment, it is worth mentioning that the shapes of formed structures are far from the modeled spheres, and thus, the values of mean size from SAXS for high-coverage samples have to be considered only as approximate ones. Meanwhile, the size of crystallites is still stable and the crystalline structure undergoes just minor changes, which are described in the following Section 3.3 in detail. These observed trends point to the coalescence sintering mechanism and indicate that the large particles observed in SEM images (Figure 3a) are, in this temperature region, still composed of multiple original seeds. Furthermore, in situ XRD measurement indicates that NPs partially melt in this temperature zone, and the volume of the crystalline phase decreases during the heating; see Figure 4a. This agrees with the findings reported in ref 17 that showed that the surface of NPs is melted and a liquid-like layer is created. For ex situ measurements, the surface solidifies again, but the recrystallization is enabled during heating.

**Figure 4:**
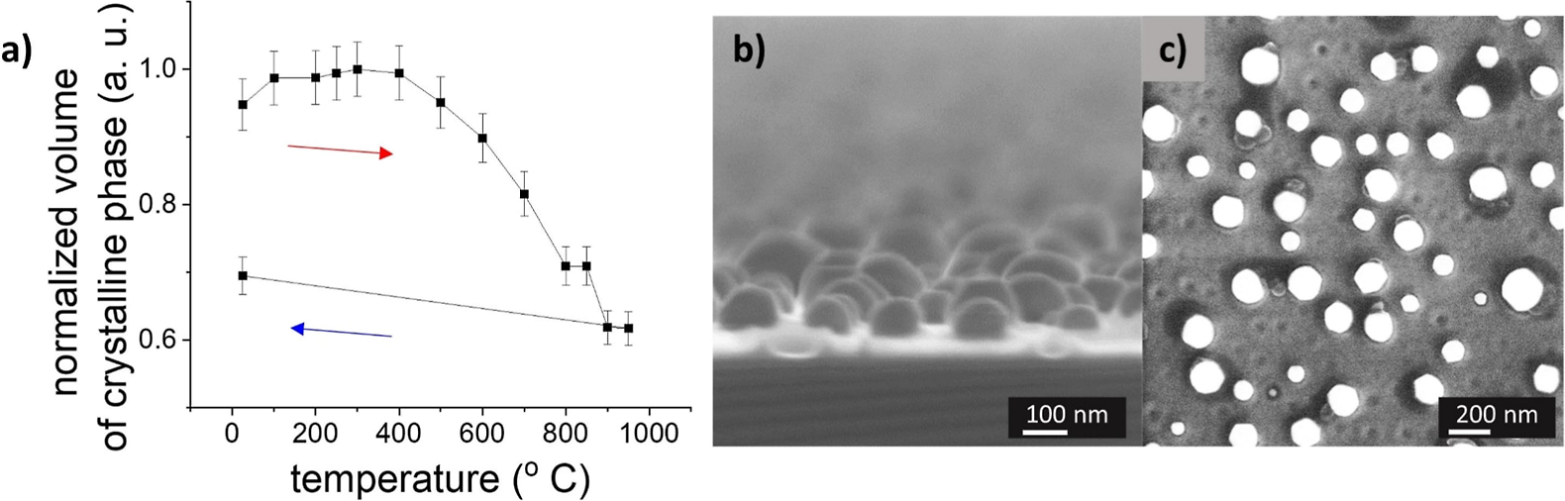
(a) Volume of crystalline phase dependent on temperature from the in situ measurement (the red arrow depicts annealing and the blue arrow depicts cooling). (b) Cross-section SEM and (c) SEM micrographs of the sample with high coverage of nanoparticles after heating to 1050 °C.

Finally, for the highest temperatures (>800 °C), a growth step in nanoparticle size occurs in the case of samples with high surface coverage of Au NPs (Figure 3c). This step results from the finished spheroidization process, i.e., the finished coalescence of the NPs. In addition, the growth of NPs in size in this temperature region is accompanied by an abrupt increase in the mean crystallite size. Taking into account the almost constant size of crystallites in the midtemperature region, this result suggests that the crystallites start to grow after the aggregation and rounding of NPs are completed. Such delayed growth of crystallites was already reported on a multilayered stack of chemically prepared NPs of diameter 10 nm.^39^ The absence of this temperature region in the case of low surface coverage by Au NPs is due to the larger distances between individual nanoparticles on the substrate that inhibit their sintering. The shift of the mean NP size to higher values occurring above 1000 °C is caused mainly by the evaporation of the smallest NPs.

It is worth stressing that after reaching 900 °C, some material is irreversibly lost due to evaporation. In fact, the crystalline volume is about 1/4 lower after annealing to approximately 950 °C followed by cooling to room temperature (Figure 4a). In addition, numerous randomly distributed holes appear on the substrate heated at high temperatures (Figure 4). Similar holes were observed in previous research^40^ during dewetting of the gold layer and nanoparticle formation at 1102 °C after annealing for 3 h. Their occurrence is explained by the formation of small initial crystals of fused silica, which crystallize above 1100 °C, as fused silica was used as a substrate in the previous study. However, this process may be ruled out in our case, as XRD measurements do not show any new crystalline phase after the annealing. In contrast, we observe the disappearance of the smallest gold nanoparticles from the samples annealed at the temperature of 1050 °C. Based on these results, we propose an alternative explanation of the formation of holes that can be summarized as follows. Au nanoparticles lie on the SiO_*x*_ layer of around 2 nm thick (measured by ellipsometry) at the beginning of the process. Due to heating, the thickness of the SiO_*x*_ layer increases up to 10 nm. Simultaneously, the gold nanoparticles tend to thermally penetrate the silicon dioxide layer.^41^ Indeed, it is possible to see nanoparticles partially sunk into the SiO_*x*_ layer on a cross-section SEM image of a sample annealed to 1050 °C (Figure 4b). This also explains the impossibility of manipulating nanoparticles by the AFM tip after annealing to 800 °C reported in the study made by Oras et al.^42^ However, as the thermal stability is dramatically lower for small NPs as compared to bigger ones, the small NPs may evaporate after annealing to 1050 °C, which results in the formation of holes in the SiO_*x*_ layer visible by AFM and SEM. This is furthermore in agreement with the irreversible reduction of the volume of the crystalline gold phase (Figure 4a).

Markedly different behavior was observed for the Au thin film. In this case, we observe a continuous dewetting leading to the formation of irregular interconnected Au structures and at higher temperatures to the appearance of individual Au particles that resemble the morphology of heated high-coverage Au nanoparticle films. However, in contrast to high-coverage Au nanoparticle films, the formed structures are in general significantly bigger and exhibit much higher size variations as compared to high-coverage Au nanoparticle films (Figure 3a). The formation of Au particles is also accompanied by a gradual increase in the size of crystallites for temperatures up to 750 °C; see Figure 3d. Above this temperature, the morphology of the Au structures as well as the mean size of crystallites remains constant.

The preferred orientation of crystallites on the substrate was investigated by pole figure measurements. As mentioned previously, the as-deposited-state NPs exhibit random orientation of crystallites, contrary to the sputtered thin layer, which possesses the {111} fiber texture. However, for NP layers, at high temperatures, the rise of the preferred orientation of the crystallites toward the substrate is detected. The (111) pole figures of the samples annealed to 1050 °C are shown in Figure 5a–c. The radii of the dotted circles in Figure 5 correspond to the angles of other crystallographic planes to the {111} planes. Hence, the texture components {111}, {110}, and {100} are clearly present on the low coverage sample. The signal is less pronounced for high coverage, but the same components can be found and confirmed by further measurements. In the case of the gold thin layer, just the {111} texture is detected.

**Figure 5:**
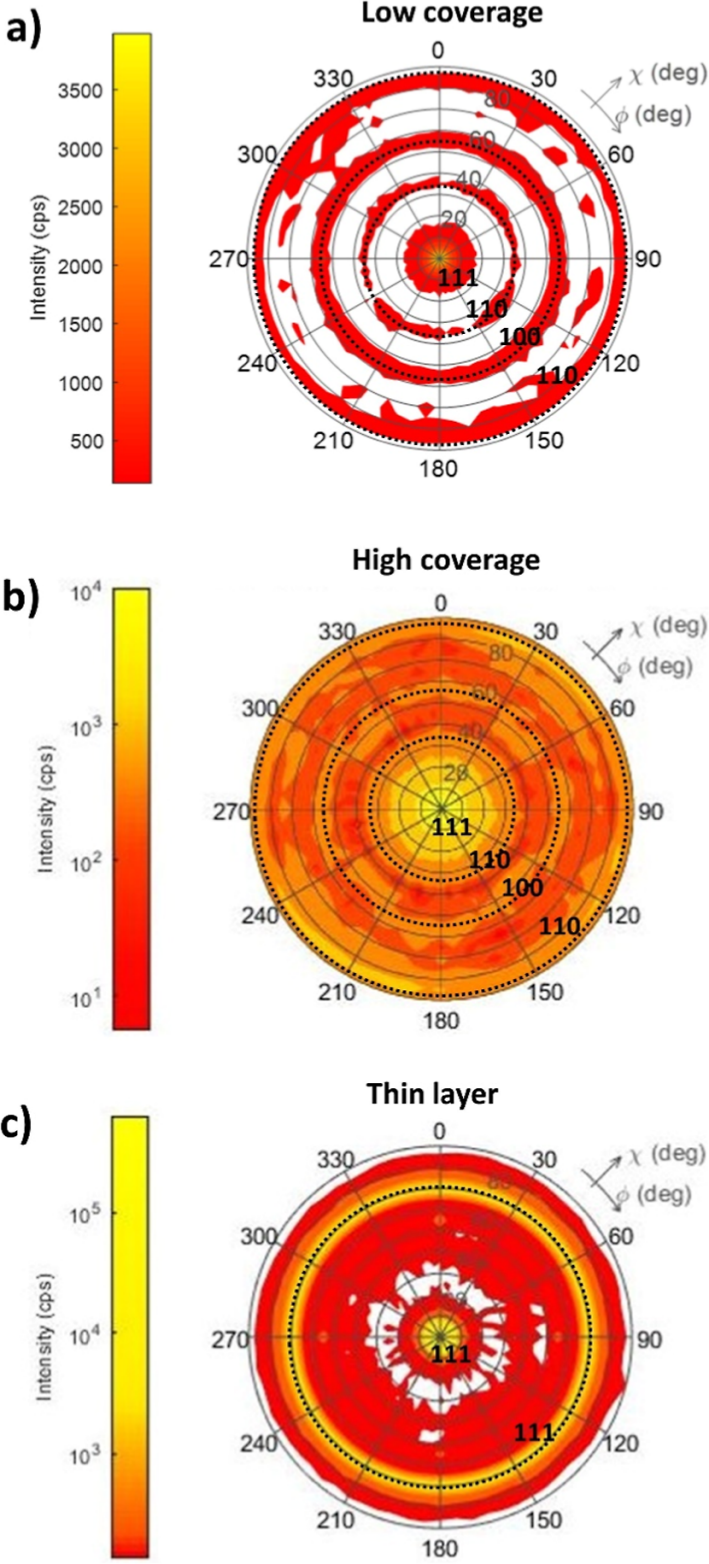
Pole figure (111) for samples annealed to 1050 °C: (a) low coverage, (b) high coverage, and (c) thin layer. The radii of the dotted circles correspond to the angles of other crystallographic planes to the {111} planes. Suggesting that planes {1 1 1} are parallel to the sample surface, the maximum at 90 deg in Figure 5a and b corresponds to planes {-1 1 0}, and its permutations and the maximum in Figure 5c at 70 deg correspond to planes {-1 1 1} and its permutations.

The texture evolution was investigated in situ by χ scans for the high-coverage sample; see Figure S2a in Supporting Information The χ scan probes the intensity during the inclination of the sample surface normal direction and, therefore, is sensitive to the presence of texture in the sample. From no preferential orientation for as-deposited NPs, the formation of {111} fiber texture is detected above 850 °C, i.e., the grains with {111} planes aligned parallel to the substrate are present in the sample more often than other orientations. This is enabled by melting significant parts of individual NPs and possible slight rotation of whole nanoparticles. Texture components arising from planes {111} and {110} aligned parallel to the surface are observed above 950 °C; for the {100} texture component, the evolution is not shown in Figure S2a due to the overlap with the substrate peak. After ex situ heating to 1050 °C, three texture components are present. The surfaces corresponding to these orientations have the lowest surface energy as the atoms on the surface are packed closest.^43^ They probably arise from the highly complex process of coalescence and self-ordering of the atoms and whole NPs during annealing. The contribution of the cooling process is eliminated, as the texture formation is observed already during the in situ measurement. All three texture types were also observed during the dewetting of gold NPs and subsequent heating on fused silica in the study made by Kracker et al.^40^ Contrary to that, the dewetted NPs in our study exhibit just the strengthening of the initial {111} texture, as clearly visible from the χ scans in Figure S2b measured ex situ after annealing. The difference in results may be caused by different substrates or shorter annealing times in our study.

### 3.3 Evolution of Structural Defects during Annealing

The evolution of microstrain and stacking fault densities with temperature for nanoparticle films with both surface coverages and a thin Au layer are depicted in Figures 6 and 7. As expected, the structural defects are gradually vanishing with increasing temperature. However, the evolution of microstrain and stacking fault densities have different courses depending on the character of the as-deposited samples.

**Figure 6:**
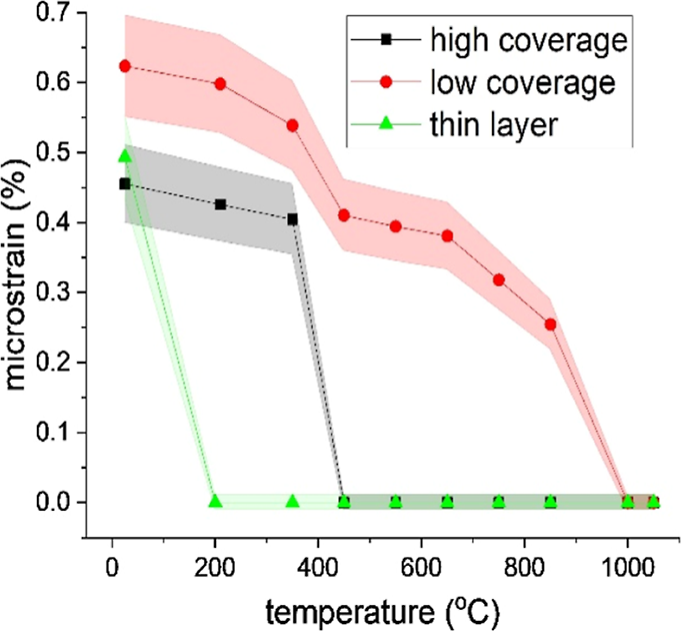
Dependence of microstrain on temperature. The standard deviations are depicted as shaded areas.

**Figure 7:**
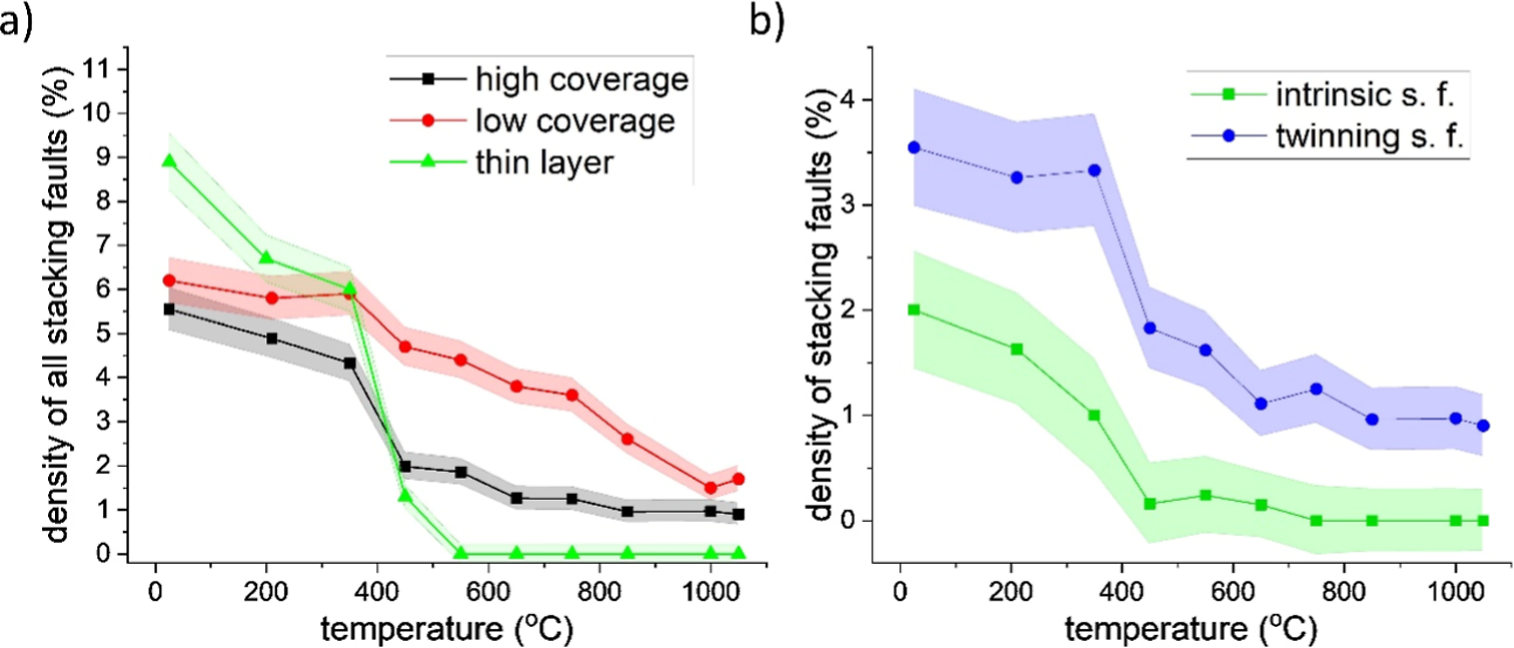
(a) Temperature dependence of stacking faults for all the samples. (b) Deformation and twinning stacking faults distinguished for high-coverage samples. The standard deviations are depicted as shaded areas.

Concerning the microstrain, whose temperature dependencies are presented in Figure 6, a temperature of around 450 °C was found to be high enough for the crystal structure to fully recover for the sample with more nanoparticles on the substrate. A similar step-like decrease of microstrain was observed around 200 °C for approximately three times smaller gold nanoparticles in a vacuum by Prymak et al.^44^ It can be observed that this temperature corresponds to the start of the aggregation process: the attachment of nearby NPs. On the other hand, for low-coverage samples, the strain decreases continually with one drop above 450 °C, i.e., the same temperature as for the high-coverage samples, but the temperature needed to fully suppress the microstrain is 1000 °C. Thus, the decrease in microstrain is tightly connected to the increase in NP size. Finally, in the case of a continuous gold layer, it is sufficient to anneal the sample to 200 °C to remove the microstrain from the crystal structure, and the interplanar spacings are equal throughout all the sample. We anticipate that the reason for different microstrain evolution in NPs and sputtered film is probably due to the different origins of the microstrain in NPs and sputtered film, respectively. In the sputtered Au layer, the microstrain arises from the ordering of the individual atoms on the substrate and the subsequent coalescence of primarily created islands, until the atoms are cold. In contrast, the nanoparticles aggregate in the aggregation chamber, and their structure is not changed much by the deposition, i.e., during their soft-landing onto a substrate. Another possible reason could be a more homogeneous distribution of the material on the substrate in the case of the sputtered gold layer.

Concerning the stacking faults, their density decreases with the increasing temperature, with a sharper drop at 450 °C, as can be seen in Figure 7a. This corresponds to the step-like decrease in the amount of microstrain. In addition, similarly to the microstrain, the decrease in the density of stacking faults is strongly dependent on the structure of the as-deposited samples: while the stacking faults completely diminish after annealing at higher temperatures in the case of sputtered Au (initial stacking fault density, 8.9%), a certain level of stacking faults is present in the nanoparticle films even after their heating to 1050 °C (around 1.2%). Furthermore, the density of stacking faults is consistently higher for low-coverage samples as compared to high-coverage ones (at the initial state 6.2% vs 5.6%, at 450 °C 4.7% vs 2.0%). Such an evolution of stacking fault densities corresponds to the structure ordering with increasing temperature and, namely, with the size growth that is different for low- and high-coverage nanoparticle films (Section 3.2).

In the case of a higher amount of NPs on the substrate, it was also possible to study the dependence of densities of twinning and deformation faults on heating temperature. As depicted in Figure 7b, both faults’ densities drop after heating to 450 °C; while the twinning faults’ density decreases to 2%, the deformation faults almost completely disappear. Another slow, gradual decrease in twinning faults is observed up to the highest temperature. The higher density and thermal stability of twinning faults are given by their lower stacking fault energy.

## 4 Conclusions

The investigation of the morphology and structure of Au nanoparticle films during their annealing in a statistically relevant manner by imaging and X-ray techniques can be summarized as follows. First of all, different phases of heat-induced morphological and microstructural changes were observed.

(i) Up to 450 °C, the Au nanoparticles undergo the Ostwald ripening process that is accompanied by an increase of the crystallite sizes for both low- and high-coverage samples as well as by the ordering of the structure for all the samples that results in a slight decrease of microstrain and density of stacking faults.
(ii) Above 450 °C, different morphogenesis were observed for low- and high-coverage samples. For the low-coverage samples, further heating above this temperature has no impact on the size/shape of Au nanoparticles and crystallite sizes. In contrast, the coalescence and spheroidization of nanoparticles take place between 450 and 850 °C for the high-coverage nanoparticle films, while the mean size of crystallites stays constant. After the completion of the spheroidization process, the increase in the temperature leads to further growth of crystallites. The number of deposited nanoparticles influences also the dependence of the microstrain and density of stacking faults on the temperature in this temperature zone: the density of stacking faults and the amount of microstrain decrease faster for high-coverage samples. It shows that the ordering of the structure depends not only on the temperature but also on the available material.
(iii) At the highest temperatures, the ordering of the nanoparticles with respect to the substrate is detected. This is accompanied by the partial penetration of Au nanoparticles into the SiO_*x*_ layer and above 1000 °C to the evaporation of small nanoparticles. This leads to the formation of holes in the SiO_*x*_ substrate.

In comparison with the parallelly performed characterization of annealed magnetron-sputtered Au thin films, it can be concluded that while the healing of the structural defects occurs at lower temperatures in the case of Au films as compared to nanoparticles, a much narrower distribution of nanoparticles can be achieved after annealing of Au nanoparticle films in comparison to the dewetting from the Au layer.

## Supplementary Material



## Data Availability

The data underlying this study are not publicly available due to their size and amount. The data are available from the corresponding author upon reasonable request.

## Notes

X-ray reflectivity on the thin Au layer, χ scans probing the intensity during the inclination of the sample surface normal direction, and goodness-of-fit (GoF) values depending on fitting parameters refined (PDF)
