# Supplementary material for: Temperature-Driven Morphological and Microstructural Changes of Gold Nanoparticles Prepared by Aggregation from the Gas Phase
Source: ACS Omega. 2025 May 20;10(21):22052–61. doi: 10.1021/acsomega.5c02149 (PMC12138619; doi:10.1021/acsomega.5c02149)
Supplement: Supplementary file 1 [file ao5c02149_si_001.pdf]

Supplementary information

Temperature-Driven Morphological and Microstructural Changes of Gold Nanoparticles Prepared by Aggregation from the Gas Phase

Tereza Košutová[1]*, Zdeněk Krtouš[2]*, Jaroslav Kousal[2], Ondřej Kylián[2], Jan Hanuš[2], Lidia Martínez[3], Yves Huttel[3], Daniil Nikitin[2], Pavel Pleskunov[2], Hynek Biederman[2], Lukáš Horák[1], Milan Dopita[1]

[1] *Department of Condensed Matter Physics, Faculty of Mathematics and Physics, Charles University, Ke Karlovu 5, 121 16 Prague, Czech Republic*

[2] *Department of Macromolecular Physics, Faculty of Mathematics and Physics, Charles University, V Holešovičkách 2, 180 00, Prague, Czech Republic*

[3] *Instituto de Ciencia de Materiales de Madrid, Consejo Superior de Investigaciones Científicas (CSIC), C/Sor Juana Inés de la Cruz, 3, 28049 Madrid, Spain*

* Corresponding authors.
E-mail address: kosutovat@gmail.com, krtousz@gmail.com

1. **Supplementary information S1 –** X-ray reflectivity on the thin Au layer

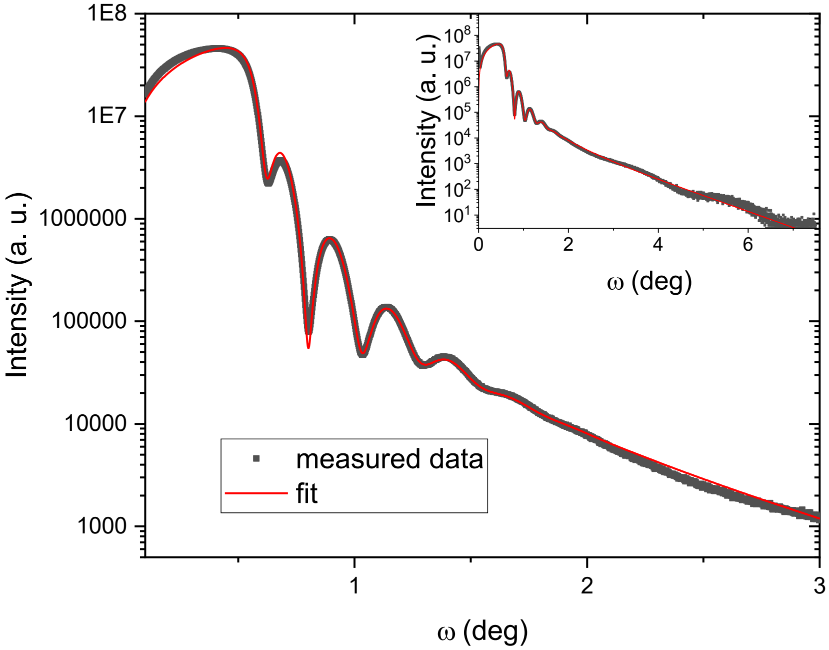

Figure S1 - Measured X-ray reflectivity for an Au layer (grey squares) and the fit (red line).

2. **Supplementary information S2 -** χ scans probing the intensity during the inclination of the sample surface normal direction, and therefore sensitive to the presence of texture in the sample

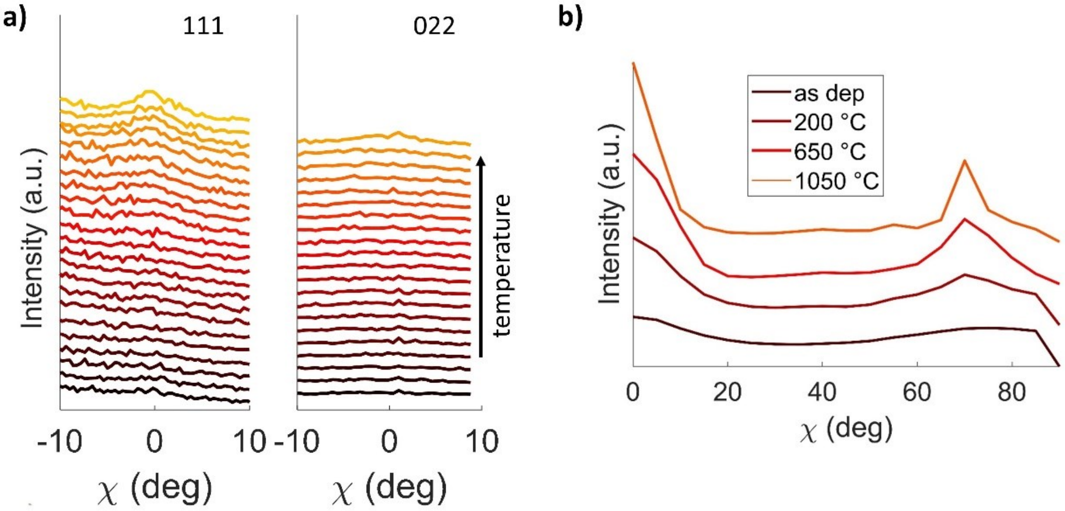

Figure S2 - χ scans a) measured in-situ during annealing of the high coverage sample, b) measured ex-situ for thin gold layer sample.

3. **Supplementary information S3** - Goodness-of-fit (GoF) values depending on fitting parameters refined

**Refined parameters - microstructural defects**	**GoF**
microstrain, twinning s. f., deformation s. f.	1.34
twinning s. f., deformation s. f.	1.96
microstrain	2.14
microstrain, twinning s. f.	1.44
microstrain, deformation s. f.	1.46

Table S1 - Goodness-of-fit (GoF) values for high-coverage sample measured after preparation at room temperature for various microstructural defect parameters included in the model (s. f. stands for stacking faults).
